# Biomarkers for the detection of renal fibrosis and prediction of renal outcomes: a systematic review

**DOI:** 10.1186/s12882-017-0490-0

**Published:** 2017-02-20

**Authors:** Sherry G. Mansour, Jeremy Puthumana, Steven G. Coca, Mark Gentry, Chirag R. Parikh

**Affiliations:** 1 Program of Applied Translational Research, Department of Medicine, Yale University, School of Medicine, New Haven, CT USA; 2 Section of Nephrology, Yale University School of Medicine, 60 Temple Street, Suite 6C, New Haven, CT 06510 USA; 3 Department of Medicine, Division of Nephrology, Icahn School of Medicine at Mount Sinai, New York, NY USA; 4 Harvey Cushing/John Hay Whitney Medical Library, Yale University, New Haven, CT USA; 5 Veterans Affairs Connecticut Healthcare System, New Haven, CT USA

**Keywords:** Fibrosis, Biomarkers, Renal biopsy, Chronic kidney disease, Renal disease progression, Outcomes

## Abstract

**Background:**

Fibrosis is the unifying pathway leading to chronic kidney disease. Identifying biomarkers of fibrosis may help predict disease progression.

**Methods:**

We performed a systematic review to evaluate the reliability of blood and urine biomarkers in identifying fibrosis on biopsy as well as predicting renal outcomes. Using MEDLINE and EMBASE, a two-stage search strategy was implemented. Stage I identified a library of biomarkers correlating with fibrosis on biopsy. Stage II evaluated the association between biomarkers identified in stage I, and renal outcomes. Only biomarkers with moderate positive correlation with fibrosis (*r* > 0.40) or acceptable area under the curve (AUC >0.65) advanced to stage II.

**Results:**

Stage I identified 17 studies and 14 biomarkers. Five biomarkers met criteria to advance to stage II, but only three were independently associated with renal outcomes. Transforming growth factor β (TGF-β) correlated with fibrosis (*r* = 0.60), and was associated with 1.7–3.9 times the risk of worsening renal function in 426 patients. Monocyte chemoattractant protein-1 (MCP-1) diagnosed fibrosis with AUC of 0.66 and was associated with 2.3–11.0 times the risk of worsening renal function in 596 patients. Matrix metalloproteinase-2 (MMP-2) correlated with fibrosis (*r* = 0.41), and was associated with 2.5 times the risk of worsening renal function.

**Conclusions:**

Given the heterogeneity of the data due to diverse patient populations along with differing renal outcomes, a meta-analysis could not be conducted. Nonetheless we can conclude from the published data that TGF-β, MCP-1 and MMP-2 may identify patients at risk for renal fibrosis and hence worse renal outcomes.

**Electronic supplementary material:**

The online version of this article (doi:10.1186/s12882-017-0490-0) contains supplementary material, which is available to authorized users.

## Background

Chronic kidney disease (CKD) prevalence and its associated healthcare costs continue to rise. The prevalence of CKD defined as estimated glomerular filtration rate (eGFR) less than 60 ml/min/1.73 m^2^ has steadily increased from 1988 to 2012, affecting over 19 million Americans [1]. Medicare costs for CKD are up to $45 billion, which is a 54% increase between 2008 and 2012 [2]. This data highlights the immense impact of CKD on socioeconomics and public health. With this increase in CKD prevalence, biomarkers to identify and predict CKD progression have been increasingly studied. There has been significant progress in biomarkers of renal injury over the past decade, with biomarkers of fibrosis recently gaining focus in the literature [3, 4]. It is important to identify and predict renal fibrosis via the use of biomarkers since tubulointerstitial fibrosis is the unifying feature in progressive renal disease irrespective of the initial insult [5]. Currently, the only clinical tool available to identify fibrosis is a kidney biopsy. However, this approach is invasive and carries certain risks, and is therefore not performed routinely [6, 7]. Identifying biomarkers of fibrosis is indispensible to the understanding of CKD progression since they can offer vital information in a noninvasive manner. Having a reliable panel of fibrosis biomarkers also has the potential to identify a subgroup of at risk patients who can be targeted for future clinical trials in hopes to improve CKD outcomes.

The objectives of this systematic review are to evaluate the reliability and performance of biomarkers of fibrosis in human studies in identifying fibrosis on biopsy and for the prediction of renal outcomes.

## Methods

### Study identification

In consultation with a research librarian, a two-step search strategy was performed to identify relevant literature. An initial search of MEDLINE and EMBASE was undertaken followed by analysis of the text words contained in the title and abstract, and of the index terms used to describe articles. A second search, using all identified keywords and index terms, was used across included databases. Lastly, the references of all identified articles were searched for any additional studies. Studies published in the English language from January 1995 to May 2016 were considered for inclusion.

The search was comprised of two stages. Stage I was constructed to identify a library of biomarkers that positively correlated with histological findings of fibrosis on biopsy. The keywords used to conduct stage I of the systematic review included ‘biological markers’, ‘markers’, ‘biomarkers’, and ‘fibrosis’ cross-referenced with ‘chronic renal insufficiency’, ‘kidney disease’ and ‘chronic kidney disease’. Stage II was aimed to evaluate the association between biomarkers in stage I and renal outcomes. To focus on the most relevant and promising biomarkers in the literature, only biomarkers with moderate positive correlation with fibrosis (*r* > 0.40) or acceptable area under the curve (AUC > 0.65) were assessed in stage II. For stage II, a separate search was conducted for the selected biomarkers and cross-referenced with the following keywords: ‘chronic kidney failure’, ‘chronic renal insufficiency’, ‘kidney diseases’, ‘kidney prognosis’, ‘renal prognosis’, ‘disease progression’, ‘renal function outcome’, ‘long term outcome’, and ‘progression of renal failure’.

### Study selection

Study eligibility for stage I included studies with patients of all ages, biomarkers that were measured either in blood or urine, and studies that included a renal biopsy as the gold standard to evaluate the level of fibrosis. Studies were excluded from stage I if fibrosis was not defined or assessed on biopsy or if only tissue biomarkers were used.

The inclusion criteria for stage II also included studies with patients of all ages and biomarkers that were measured in blood or urine but studies were only eligible if biomarker measurement preceded renal outcomes. Studies included in stage II had to have at least one outcome as worsening of renal function defined histologically or by a change in urinary albumin or protein excretion, serum cystatin-C, serum creatinine or eGFR. Studies that included patients on renal replacement therapy at enrollment or studies that only assessed tissue biomarkers were excluded from stage II. Also studies that assessed composite outcomes of renal and non-renal events without evaluating the sole association of the biomarker with the renal event were excluded. All studies included were required to have a statistically significant adjusted point estimate or AUC associating the biomarker with the specified renal outcome.

### Data collection and abstraction

Data was obtained using a standardized data extraction tool. For both stages, the data extracted included details regarding the biomarker used, the type of patient population, and sample size. Specifically for stage I, we also included the grading system used to define fibrosis on kidney biopsy as well as a Pearson correlation coefficient or sensitivity, specificity and AUC when available. For stage II, the data collection included length of follow-up for each study, as well as the study’s defined renal outcome and point estimate with 95% confidence interval or sensitivity, specificity and AUC if available.

### Quality assessment

Two independent reviewers assessed the papers selected. The methodological validity for studies included in stage II was assessed using standards for reporting diagnostic accuracy studies (STARD) criteria [8]. Out of the 25 STARD criteria, we used the ten most relevant parameters to assess quality for this review since the studies included are mainly prognostic rather than diagnostic in nature (Additional file 1) [9]. Studies with a score ≥9 were designated as ‘good’ quality, 7–8 as ‘fair’ quality and ≤6 as ‘poor’ quality. Any disagreements that arose between the reviewers were resolved through discussion, or if necessary, by referral to a third reviewer.

## Results

**Fig. 1:**
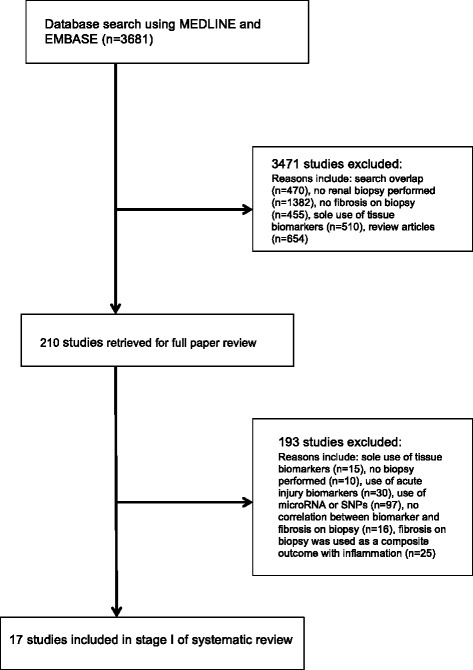
Identification process for eligible studies for stage I

**Fig. 2:**
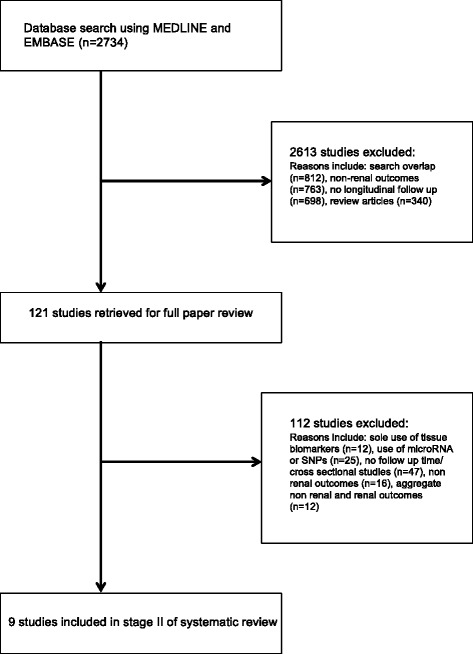
Identification process for eligible studies for stage II

The literature search for stage I identified 3681 published articles since January 1995, of which 3471 were excluded upon title and abstract review (Fig. 1). Of the remaining 210 articles, only 17 were included in stage I [10–26]. In stage II, a total of 2734 articles were identified, from which 121 were selected for full-text evaluation (Fig. 2). From these, 9 studies were eligible to be included in stage II [27–35].

### Stage I study characteristics

Fourteen distinct blood and urine biomarkers were evaluated in 2378 patients across the 17 studies identified in stage I (Tables 1 and 2). The studies assessed heterogeneous patient populations, which included patients with IgA nephropathy, lupus nephritis, anti-neutrophil cytoplasmic antibody (ANCA) vasculitis, idiopathic membranous glomerulonephritis, and renal transplant recipients. In all studies, the biomarkers were evaluated for the primary outcome of fibrosis on biopsy. Fibrosis was evaluated by different classifications including the Oxford classification, Banff criteria, Lee’s classification, image digitalization, chronic allograft damage index (CADI) score, morphometric analysis, and semi-quantitatively (Additional file 2) [36–39]. Out of the 14 biomarkers identified, only five (36%) biomarkers had at least moderate positive correlation with fibrosis (*r* > 0.40) or acceptable AUC >0.65.

**Table 1:** Performance of biomarker correlation with fibrosis on renal biopsy

Reference	Biomarker	Patient Population	*N* (%)*	Grading of fibrosis on biopsy	Time from biomarker evaluation to biopsy	Correlation Coefficient	AUC/Specificity/Sensitivity
Correlation Coefficient r >0.40 or AUC >0.65							
El Ghoul et al [14]	Urine PIIINP	Biopsies for clinical reasons	118	Banff 2003/ Semi-quantitatively	Within 1 month	*r* = 0.32 *p* < 0.001	NR
Teppo et al [15]	Urine PIIINP	Transplant recipients	79	Banff 1997/ Semi-quantitatively	Same time^a^	*r* = 0.41 *p* < 0.001	To predict no fibrosis urine PIIINP <100 ng/mmol NR/84%/83%
Soylemezoglu et al [16]	Urine and blood PIIINP	Biopsies for clinical reasons	40	Morphometric analysis	Same time	Urine PIIINP *r* = 0.51 *p* < 0.01 Blood PIIINP *r* = 0.49 *p* < 0.01	NR
Honkanen *et al* [20]	Urine TGF- β	Idiopathic membranous glomeruloneph-ritis	27	Semi-quantitatively	Same time and 1 year prior to biopsy^b^	*r* = 0.29 *p* = 0.2 (same time) *r* = 0.86 *p* = 0.01 (1 year prior)	NR
Susianti et al [21]	Urine TGF- β	Lupus nephritis	58(76)	Semi-quantitatively	Same time	*r* = 0.60 *p* < 0.001	0.90/85%/84%
Murakami et al [22]	Urine TGF-β	Glomerulopat-hy	42	Semi-quantitatively	Same time	NR	NR
Zhang et al [24]	Urine MCP-1 Urine Hepcidin Urine LFABP	Lupus nephritis	61	Semi-quantitatively	Within 24 h	NR NR NR	0.66/59%/65%
							0.48/93%/35%
							0.60/85%/41%
Chang et al [25]	Blood PAI-1	Transplant recipients	50	CADI score	Same time	*r* = 0.41 *p* = 0.003	NR
Sanders et al [26]	Urine MMP-2 Urine TIMP-1	ANCA vasculitis	29	Semi-quantitatively	Same time	Urine MMP-2 *r* = 0.41 *p* = 0.02 Urine TIMP-1 *r* = 0.35 *p* = 0.05	NR
Correlation Coefficient r ≤0.40 and AUC ≤0.65							
Grenzi et al [18]	Blood CD30	Transplant Recipients	511(25)	Banff 2007	10 days to 9.8 years	NR	NR
Amer et al [10]	Urine RBP	Transplant recipients	221(36)	Banff 1997	Same time	*r* = 0.20 *p* = 0.003	NR
Barbosa de Deus et al [11]	Urine RBP	Glomerulopa-thy	100	Image digitalization	Same time	NR	NR
Pallet et al [12]	Urine RBP	Biopsies for clinical reasons	162	Numerical quantification software	Same time	*r* = 0.33 *p* = 0.001	Fibrosis score > 25% NR/95%/20%
Zhu et al [13]	Blood VCAM-1	IgA nephropathy	327(23)	Oxford classification	Same time	NR	NR
Metalidis et al [17]	Urine CTGF	Transplant recipients	315^1^ 225	Banff 1997	Same time and 21 months apart	NR	Same time AUC = 0.63 21 months: AUC = 0.65
Liu et al [19]	Urine MBL	IgA nephropathy	162	Lee’s classification/ Oxford classification	Same time	NR	NR
Lu et al [23]	Urine SGK-1	IgA nephropathy	76	Oxford classification	Same time	*r* = 0.24 *p* = 0.04 (adjusted for M)^c^ *r* = 0.37 *p* = 0.001 (adjusted for E)^c^ *r* = 0.34 *p* = 0.003 (adjusted for S)^c^	NR

*ANCA* anti-neutrophil cytoplasmic antibodies, *AUC* area under the curve, *CADI* chronic allograft damage index, *CTGF* connective tissue growth factor, *LFABP* liver-type fatty acid-binding protein, *MBL* mannose-binding lectin, *MCP-1* monocyte chemoattractant protein-1, *MMP-2* matrix metalloproteinase-2, *NR* not reported, *PAI-1* plasminogen activator inhibitor 1, *PIIINP* amino-terminal propeptide of type III procollagen, *RBP* retinol- binding protein, *SGK-1* serum- and glucocorticoid-inducible kinase, *TIMP-1* tissue inhibitor of metalloproteinase-1, *TGF- β* transforming growth factor-beta, *VCAM* vascular cell adhesion molecule 1

* When provided by study, ‘*N*’ represents the total number of participants receiving a biopsy and (%) is those with fibrosis on biopsy

^1^315 had CTGF level drawn same time as biopsy, but only 225 had CTGF drawn 21-month prior to biopsy

^a^Some biopsies were done a few days apart from biomarker measurement

^b^7 patients had biomarker measured 1 year prior to biopsy

^c^ *M* mesangial hypercellularity, *S* segmental glomerulosclerosis, *E* endocapillary hypercellularity

**Table 2:** Characteristics of studies included in stage I

Reference	Biomarker	Age (years) Mean ± SD Median (range)	Sex (% Male)	Race (% Caucasian)	Method used to obtain GFR	Baseline GFR Mean ± SD Median (range)
El Ghoul et al [14]	Urine PIIINP	46 ± 17	48%	88%	Four variable MDRD	48.9 (3.4 - 203.1)
Teppo et al [15]	Urine PIIINP	47 (22-68)	61%	NR	24-h creatinine clearance	56.2
Soylemezoglu et al [16]	Urine and blood PIIINP	51 ± 18	NR	NR	NR	NR
Honkanen et al [20]	Urine TGF- β	43^a^	65%	NR	51Cr-EDTA- clearance or 24-h creatinine clearance	NR
Susianti et al [21]	Urine TGF- β	30^b^	7%	NR	NR	NR
Murakami et al [22]	Urine TGF-β	NR	NR	NR	NR	NR
Zhang et al [24]	Urine MCP-1, Hepcidin, LFABP	30 (17-51)	11%	46%	NR	NR
Chang et al [25]	Blood PAI-1	51^a^	10%	NR	MDRD	31.5^c^
Sanders et al [26]	Urine MMP-2 Urine TIMP-1	67 (23-86)	NR	NR	NR	NR
Grenzi et al [18]	Blood CD30	35 (4.8–67.1)	56%	29%	Cockgraft-gault	NR
Amer et al [10]	Urine RBP	52 ± 13	55%	93%	Four variable MDRD Iothalamate measurement	53.7 ± 14.9 57.5 ± 17.1
Barbosa de Deus et al [11]	Urine RBP	33 ± 12	54%	53%	Creatinine clearance	73.2 (33-172)^d^ 97.5 (45-175)
Pallet et al [12]	Urine RBP	53 ± 18	54%	53%	MDRD	47.4 ± 33.3
Zhu et al [13]	Blood VCAM-1	33 ± 11	47%	NR	NR	85.4 ± 30.3
Metalidis et al [17]	Urine CTGF	53 ± 13.2	61%	NR	MDRD	53.3 ± 17.4
Liu et al [19]	Urine MBL	35	57%	NR	Four variable MDRD	85.9
Lu et al [23]	Urine SGK-1	38^b^	52%	NR	MDRD	90.8 ± 43.2

*CTGF* connective tissue growth factor, *GFR* glomerular filtration rate, *LFABP* liver-type fatty acid-binding protein, *MBL* mannose-binding lectin, *MCP-1* monocyte chemoattractant protein-1, *MMP-2* matrix metalloproteinase-2, *NR* not reported, *PAI-1* plasminogen activator inhibitor 1, *PIIINP* amino-terminal propeptide of type III procollagen, *RBP* retinol- binding protein, *SD* standard deviation, *SGK-1* serum- and glucocorticoid-inducible kinase, *TIMP-1* tissue inhibitor of metalloproteinase-1, *TGF- β* transforming growth factor-beta, *VCAM* vascular cell adhesion molecule

^a^This age was obtained by taking the average of the median ages

^b^This age was obtained by taking the average of the mean ages

^c^This GFR represents the average of the medians

^d^The top GFR represents patients with abnormal RBP and the bottom GFR represents patients with normal RBP

### Stage I biomarker performance (Tables 1 and 2)

*Amino-terminal propeptide of type III procollagen* (PIIINP) was evaluated in three studies, encompassing a total of 237 patients with a mean age ranging from 46 to 51 years [14–16]. Overall, blood and urine PIIINP had moderate positive correlations with fibrosis on biopsy with Pearson coefficients ranging from *r* = 0.32 to *r* = 0.51. Using Banff 1997 criteria and semi-quantitative methods to assess fibrosis on biopsy, urine PIIINP positively correlated with fibrosis (*r* = 0.41, *p* < 0.001) and lower levels of PIIINP predicted no fibrosis with a specificity of 84%, a sensitivity of 83%, and a positive predicative value (PPV) of 81% [15]. Another study used morphometric analysis to assess fibrosis and found that both urine and blood PIIINP positively correlated with fibrosis (*r* = 0.51, *p* < 0.01 and *r* = 0.49, *p* < 0.01, respectively) [16].

*Transforming growth factor beta* (TGF-β) was assessed in three studies, encompassing a total of 127 patients with mean age ranging from 30 to 43 years [20–22]. Fibrosis on biopsy was assessed semi-quantitatively in all three studies. Urine TGF-β positively correlated with fibrosis on biopsy (*r* = 0.60, *p* < 0.001), and was able to diagnose fibrosis >5% with an AUC of 0.90 [21]. Urine TGF-β was also found to positively correlate with future fibrosis on biopsy in seven patients 1-year post biomarker measurement (*r* = 0.86, *p* = 0.01) [20].

*Monocyte chemoattractant protein* (MCP-1) was evaluated in 61 patients with lupus nephritis and a median age of 30 years [24]. Biopsies were done within 24 h of urine biomarker measurement and fibrosis on biopsy was assessed semi-quantitatively. Urine MCP-1 was able to diagnose fibrosis on biopsy with an AUC of 0.66.

*Plasminogen activator inhibitor-1* (PAI-1) was evaluated in 50 renal transplant patients with a mean age of 51 years and baseline eGFR of 32 ml/min/m^2^ [25]. The CADI score was used to quantify fibrosis on biopsy. Blood PAI-1 levels positively correlated with fibrosis on biopsy (*r* = 0.41, *p* = 0.003).

*Matrix metalloproteinase-2* (MMP-2) was assessed in 29 patients with ANCA vasculitis and a median age of 67 years. Using a semi-quantitative method to measure fibrosis on biopsy, urine MMP-2 positively correlated with fibrosis on biopsy with *r* = 0.41.

### Stage II study characteristics

Out of the five biomarkers identified in stage I to have at least *r* > 0.40 or AUC > 0.65, only three, TGF-β*,* MMP-2, and MCP-1, were independently associated with renal outcomes over longitudinal follow-up. A total of nine articles were included in stage II (Table 3 and 4). The studies assessed different patient populations, which included patients with type II diabetes, obstructive nephropathy, those receiving coronary angiography, renal transplant patients and simultaneous pancreas and kidney transplant patients. In all studies, the biomarkers were independently associated with worsening renal function.

**Table 3:** Associations between stage II biomarkers and renal outcomes

Reference	Biomarker/cut off	Patient Population	Subjects *N* (%)*	Definition of renal progression	Follow up: Median (range) Mean (± SD)	Point Estimate (95% CI; *p*-value)	C-statistic or AUC/specificity/sensitivity	Quality Score
Chen et al [29]	Urine TGF-β > 569 ng/l	Unilateral ureteral obstruction requiring percutaneous nephrostomy	45 (24)	Non-functioning kidney group defined by no improvement in eGFR	3 months	NR	NR/82%/82%	**Fair (7)**
Harris et al [30]	Blood TGF- β	Renal transplant recipients	100 (23)	Biopsy proven chronic allograft nephropathy using Banff 97	5 years	HR 1.7 (1.1-2.6; *p* = 0.008)	NR/NR/NR	**Good (10)**
Wong et al [31]	Blood total TGF- β 1 Blood active TGF- β 1	Type II diabetes	102 179 (HC)	Doubling of serum creatinine	5 years	Blood total TGF- β 1: OR 3.9 (2.1-7.3)	Conventional predictors:^a^ 0.75/NR/NR	**Fair (7)**
						Blood active TGF- β 1: NR	Addition of total TGF-β1: 0.82/NR/NR	
							Addition of active TGF-β1: 0.88/NR/NR	
							Addition of both active and total TGF- β 1: 0.96/NR/NR	
Hsu et al [27]	Blood MMP-2	Non diabetic patients referred for coronary angiography	251 (16)	eGFR decline >25% from baseline	8.5 (±2.4) years	HR 2.5 (1.2-5.1)	NR/NR/NR	**Good (9)**
Shi et al [28]	Urine MMP-2	Chronic tubulointerstitial nephropathy	61 20 (HC)	Continuous outcome of eGFR decline	38 (11-54) months	β coefficient -0.1 ml/min/m^2^, (*p* = 0.05)	0.74 (*p* < 0.05)/NR/NR	**Fair (7)**
Titan et al [32]	Urine MCP-1 ≥ 52 ng/g	Macroalbuminuric type II diabetes	56 (27)	Composite outcome of risk of dialysis, or doubling of serum creatinine or death^b^	30.7 (±10) months	OR 11.0 (1.6–76.4; *p* = 0.02)	0.65 (*p* = 0.08)/NR/NR	**Fair (8)**
Verhave et al [33]	Urine MCP-1 ≥ 48 ng/mmol	Diabetic nephropathy	83	The rate of eGFR decline as a continuous outcome	2.1 years	β coefficient -2.0 ml/min/m2 (*p* = 0.001)	NR/NR/NR	**Fair (7)**
Ogliari et al [34]	Blood donor MCP-1 > 66^th^ percentile	SPK recipients	77	Graft loss	87.4 (65.4–132.3) months	HR 4.5 (1.2–16.8; *p* = 0.02)	NR/NR/NR	**Poor (6)**
Nadkarni et al [35]	Urine MCP-1 (continuous and tertiles)	Type II diabetes	380 (50)	eGFR decline >40% from baseline	5 years	OR (continuous) 2.3 (1.4-3.6) OR (3^rd^ *vs.* 1^st^ tertile) 5.3 (2.2-12.7)	C-statistic Conventional predictors: ^c^ 0.70 Addition of MCP-1: 0.74	**Fair (7)**

*AUC* area under the curve, *CAN* chronic allograft nephropathy, *eGFR* estimated glomerular filtration rate, *HC* Healthy controls, *HR* Hazard ratio, *MMP-2* matrix metalloprotinease-2, *MCP-1* monocyte chemoattractant protein-1, *NR* not reported, *OR* odds ratio, *PIINP* procollagen type III amino-terminal pro- peptide, *SPK* simultaneous pancreas kidney transplant, *TGF-β* Transforming growth factor-beta

*N represents the total sample size and the percentage represents the percent of those with the outcome when available in the literature

^a^Conventional Predictors: sex, body mass index, age, duration of diabetes mellitus, hemoglobin A1c, eGFR(CKD-EPI), randomized treatment interventions, urinary albumin/creatinine ratio, and history of macrovascular and microvascular events. ^b^Death only occurred in two people, hence the composite outcome was mainly worsening of renal function and study was included in phase II. ^c^ Sex, body mass index, hemoglobin A1C, eGFR, fibrate intervention, Angiotensin converting enzyme inhibitors/angiotensin II receptor blockers, urine albumin-creatinine ratio, and cardiovascular disease history

**Table 4:** Characteristics of Studies Included in stage II of the review

Reference	Biomarker	Multicenter *vs*. single center	Age (years) Mean ± SD Median (range)	Sex (% Male)	Race (% Caucasian)	Method used to obtain GFR	Baseline GFR or serum creatinine Mean ± SD Median (range)
Chen et al [29]	Urine TGF-β	Single centered	64^a^	62%	NR	99mTc-DTPA dynamic renal scintigraphy	6.7 ± 1.7 ml/min/m^2^ ^c^ 11.9 ± 2.4 ml/min/m^2^ ^d^
Harris et al [30]	Blood TGF-β	Single centered	43^b^	60%	85%	NA	2.5 mg/dl^e^ 2.1 mg/dl^f^
Wong et al [31]	Blood total TGF-β Blood active TGF-β	Multi-centered (25 international centers)	69 ± 7 69 ± 7 (HC)	70%	NR	CKD EPI	55.1 ± 19.8 ml/min/m^2^ 70.7 ± 15.8 ml/min/m^2^ (HC)
Hsu et al [27]	Blood MMP-2	Single centered	67^a^	86%	NR	CKD-EPI	73.6 ± 15.3 ml/min/m^2^
Shi et al [28]	Urine MMP-2	Single centered	46^a^ 51 ± 10.2 (HC)	31%	NR	CKD-EPI	34.3 ml/min/m^2g^ 72.2 ± 10.6 ml/min/m^2^ (HC)
Titan et al [32]	Urine MCP-1	Single centered	58 ± 10.2	63%	41%	24-h creatinine clearance	45.2 ± 22.7 ml/min/m^2^
Verhave et al [33]	Urine MCP-1	Multi-centered (4 different hospitals)	69 ± 10	80%	87%	4 variable MDRD	25.0 ± 9.0 ml/min/m^2^
Ogliari et al [34]	Blood MCP-1	Single centered	38 ± 7.2 (r) 28 ± 9 (d)	57%	NR	NA	7.9 ± 3.4 mg/dl (r)^h^ 0.9 ± 0.3 mg/dl (d)
Nadkarni et al [35]	Urine MCP-1	Multi-centered	Controls 61.9 ± 5.4 Cases 62.3 ± 5.6	Controls 51% Cases 52%	Cases and controls 74%	CKD EPI	Controls 90.2 ml/min/m^2^ Cases 87.0 ml/min/m^2^

*CKD-EPI* chronic kidney disease epidemiology collaboration, *d* donor, *DTPA* diethylenetriaminepentaacetic acid, *GFR* glomerular filtration rate, *HC* Healthy controls, *MDRD* Modification of Diet in Renal Disease, *MMP-2* matrix metalloprotinease-2, *MCP-1* monocyte chemoattractant protein-1, *NR* not reported, *r* recipient, *SD* Standard deviation, *TGF-β* Transforming growth factor-beta

^a^This age was obtained by taking the average of the mean ages

^b^This age represents the average of the mean ages of participants at time of transplant

^c^Pre-operative GFR in obstructed kidney

^d^Post-operative GFR after percutaneous nephrostomy

^e^Serum creatinine at transplant

^f^ Serum creatinine 1 month post transplant

^g^This GFR represents the average of the means

^h^Recipients were on dialysis for 4.0 ± 2.4 years

### Stage II biomarker performance (Table 3 and 4)

*Urine TGF-β* concentrations were evaluated in 426 patients with an average age ranging from 43 to 69 years across three different studies [29–31]. One study was ‘good’ quality and two were ‘fair’ quality. Higher TGF-β concentrations were associated with biopsy proven chronic allograft nephropathy in transplant recipients over a 5-years follow-up, as well as worsening renal function in patients with obstructive uropathy and type II diabetes (point estimates ranging from 1.7 to 3.9). The addition of blood TGF-β to conventional predictors such as age, sex, duration and severity of diabetes, eGFR and albumin/creatinine ratio in patients with baseline eGFR of 55 ml/min/m^2^ increased the AUC from 0.75 to 0.96 for predicting doubling of serum creatinine over a 5-years follow-up period in a nested case-control study from the ADVANCE clinical trial cohort [31].

*Blood and urine MMP-2* concentrations were measured in 332 patients across two studies with a mean age ranging from 46 to 67 years [27, 28]. One study was ‘good’ quality and another was ‘fair’ quality. Studies revealed that higher MMP-2 concentrations are associated with decline in eGFR in patients with and without CKD with baseline eGFR of 34 ml/min/m^2^ and 74 ml/min/m^2^, respectively. In one study assessing patients’ eGFR post coronary angiography over an 8 years follow up, 39 (16%) of non-CKD and non-diabetic patients had over a 25% reduction in their eGFR from baseline [27]. Those with higher blood MMP-2 levels were 2.5 times as likely to develop decline in their eGFR compared to those with lower levels. Urine MMP-2 in another study was able to predict eGFR decline with an AUC of 0.74, with a decline of 0.1 ml/min/m^2^ in eGFR for every unit increase of urine MMP-2 over a 38 month follow up [28].

*Urine MCP-1* was evaluated in a total of 596 patients in four studies with a mean age ranging from 38 to 69 years [32–35]. Three studies were of ‘fair’ quality and one was ‘poor’ quality. Over a follow-up period of 2 to 7 years, higher levels of urine and blood MCP-1 were significantly and independently associated with future eGFR decline and doubling of creatinine in native kidneys and graft loss in transplant recipients with baseline eGFR ranging from 25 ml/min/m^2^ to 90 ml/min/m^2^ (point estimates ranging from 2.3 to 11.0).

## Discussion

The relentless progression of renal disease is closely linked to the process of fibrosis, which is triggered by initial or ongoing injury. Although it is still a point of debate, there is literature to support a mechanistic rather than merely an associative role of fibrosis in progression of kidney disease [40]. This systematic review is a comprehensive evaluation of renal biomarkers that can be used in the detection of fibrosis as well as in the prediction of progression of renal disease. However, the development of a clinically useful biomarker is a sequential process that usually requires five phases; phase 1 identifies promising directions in preclinical studies, phase 2 is clinical assay validation and detection of established disease, phase 3 is biomarker prediction of clinical disease in longitudinal studies, phase 4 is prospective screening and finally phase 5 is impact of screening on disease burden [41]. This systematic review aimed to identify fibrosis biomarkers that achieved phase 2 and phase 3 of development. A total of 14 biomarkers were identified in phase 2 of development and were linked to fibrosis on biopsy, but the majority (64%) had weak correlations or unreported associations in the literature. Only five biomarkers (PAI-1, PIIINP, MMP-2, TGF-β, and MCP-1) had at least moderate correlations with fibrosis on biopsy, out of which only three (MMP-2, TGF-β, and MCP-1) were independently associated with worsening renal function.

TGF-β had the strongest correlation with fibrosis on biopsy and was significantly associated with worse renal outcomes in the literature. This is supported by strong biological plausibility in animal literature, where the overexpression of TGF-β by renal tubular epithelial cells led to tubulointerstitial fibrosis and the blocking of TGF-β ameliorated this process [42, 43]. In this review, MCP-1 had a very strong association with progression of renal disease, which is reinforced by preclinical studies showing that the blockade of MCP-1 receptor (CCR2) reduces interstitial fibrosis [44]. Lastly, MMP-2 was also a strong independent predictor of declining eGFR, which is again corroborated by decreased fibrosis in MMP-2 knockout mice [45].

However, this systematic review highlights the limitations in the available literature assessing fibrosis biomarkers. First, all three biomarkers were evaluated in both blood and urine in stage II studies but were only evaluated in urine in stage I studies. This highlights the need for further studies evaluating the correlation of blood levels of these biomarkers and fibrosis on biopsy. Also, generalizability to all patients is limited as most studies in both stages I and II evaluated specific patient populations such as lupus nephritis or IgA nephropathy. Biomarker performance differed across different patient populations, which further hinders the application to a broad patient population.

Statistical deficiencies also existed among studies. In stage I, five out of 17 studies did not report estimates of diagnostic accuracy or measures of statistical uncertainty (Table 1). Only three studies reported AUC values for diagnosing the presence and the severity of fibrosis. Lastly, stage I studies lacked a standard method to assess fibrosis with up to eight different classifications utilized (Additional file 2). This heterogeneity in the assessment of fibrosis makes it difficult to make standardized comparisons among biomarkers of fibrosis across studies.

Using the adjusted STARD quality score to assess stage II studies, only two out of nine studies were of good quality, highlighting potential areas of improvement. The majority of studies utilized convenience sampling, which introduces ‘selection bias’ as the participant sampling might not be an accurate representation of the population. Only one out of the nine studies in stage II stated that the examiners of the index test and reference standard were blinded. Lack of blinding could have introduced ‘review bias,’ as the reviewers were aware of the reference test result. The adjustment for confounding was also limited in most stage II studies lacking the current clinical gold standard to assess patient outcomes, which is the use of baseline eGFR and proteinuria (Table 5). Lastly, PAI-1 and PIIINP studies performed well in stage I, but were not included in stage II secondary to lack of longitudinal studies and lack of independent association with CKD progression after adjusting for eGFR and proteinuria, respectively [46].

**Table 5:** Stage II variables used for multivariable analyses

Reference	Biomarker	Patient Population	Variables used for multivariable analyses
Chen et al [29]	Urine TGF- β	Unilateral ureteral obstruction requiring percutaneous nephrostomy	NA
Harris et al [30]	Blood TGF- β	Renal transplant recipients	Acute cellular rejection
Wong et al [31]	Blood total and active TGF- β 1	Type II diabetes	Sex, age, baseline eGFR, randomized treatment interventions^a^, urinary albumin/creatinine ratio, hemoglobin A1c, BMI, diabetes duration, and history of macrovascular or microvascular disease
Hsu et al [27]	Blood MMP-2	Non diabetic patients referred for coronary angiography	Age, sex, smoking status, BMI, systolic blood pressure, fasting glucose, total cholesterol, and baseline eGFR
Shi et al [28]	Urine MMP-2	Chronic tubulointerstitial nephropathy	Age, baseline eGFR, mean blood pressure
Titan et al [32]	Urine MCP-1	Macroalbuminuric type II diabetes	Baseline creatinine clearance, baseline 24 h proteinuria, and systolic blood pressure
Verhave et al [33]	Urine MCP-1	Diabetic nephropathy	Proteinuria, TGF-B
Ogliari et al [34]	Blood MCP-1	SPK recipients	Hemoglobin A1c, years of dialysis pre transplant, recipient BMI, enteric drainage, >1 episode of rejection, type of immunosuppression
Nadkarni et al [35]	Urine MCP-1	Type II diabetes	Hemoglobin A1c, mean arterial pressure, history of cardiovascular disease, intensive glycemic and blood pressure control, fibrates, angiotensin receptor blockers, angiotensin converting enzyme inhibitors, thiazolidinedione, baseline eGFR, urinary albumin-creatinine ratio.

*BMI* Basic metabolic panel, *eGFR* estimated glomerular filtration rate, *HC* Healthy controls, *HR* Hazard ratio, *MMP-2* matrix metalloprotinease-2, *MCP-1* monocyte chemoattractant protein-1, *NA* not applicable, *SPK* simultaneous pancreas kidney transplant, *TGF-β* Transforming growth factor-beta

^a^Wong et al was an ancillary study from the ADVANCE trial cohort, which randomized participants to intensive glucose control, targeting a hemoglobin A1c of ≤6.5%, or to standard, guideline-based glucose control, as well as to combination perindopril–indapamide therapy or to matching placebo

We also acknowledge some of the limitations to our approach. We allowed for the liberal inclusion of all patient populations as well as a wide spectrum of renal outcomes to be able to capture the maximum number of biomarkers of fibrosis assessed in the literature. However, this approach led to heterogeneity in the data and did not allow the summation of the results into a meta-analysis (Additional file 3). In addition, our two-stage approach allowed for the selection of biomarkers that both correlated with fibrosis on biopsy and were associated with renal outcomes. Hence, only biomarkers that were both diagnostic of fibrosis and predictive of outcomes were included in this systematic review. The purpose of this design was to specifically identify biomarkers of fibrosis rather than the general identification of biomarkers of progression, but this would undervalue a good predictive biomarker that has not yet been studied in biopsy confirmed renal fibrosis.

## Conclusion

Despite the above limitations, there are promising considerations that are highlighted in this review. This review identifies gaps in the literature in the field of renal fibrosis and emphasizes the need for additional studies utilizing biopsies to identify subclinical fibrosis. Furthermore, three promising biomarkers are featured in this review to have diagnostic and prognostic potential in patients with renal disease. MMP-2, MCP-1 and TGF-β have been shown to identify patients with fibrosis and future poor renal outcomes. Since biomarkers of fibrosis have the potential to identify at risk populations as well as offer insight into possible therapeutic measures, it is imperative for future studies to evaluate the role of these biomarkers in diagnosing established interstitial fibrosis as well as evaluating their associations with future renal outcomes.

## Additional files

Additional file 1: Study quality scoring system for stage II. *Out of the 25 standards for reporting diagnostic accuracy studies (STARD) criteria, we used the ten most relevant parameters to assess quality of studies listed in this review. Studies meeting each criterion are listed under comments on the far right of the table. (DOCX 14 kb)*

Additional file 2: Methods of fibrosis assessment on biopsy in stage I studies. *The table above shows the different methods used to assess fibrosis across different studies in stage I. Fibrosis was evaluated using Banff criteria, image digitalization, numerical quantification score, Oxford classification, morphometric analysis, Lee’s classification, chronic allograft damage index (CADI) score, and semi-quantitatively. (DOC 31 kb)*

Additional file 3: Patient populations and renal outcomes assessed in stage I and stage II of the systematic review. *The above table shows the heterogeneity in the data with varying patient populations and different operational definitions of worsening renal function. (DOC 43 kb)*

## Abbreviations

ANCA: Anti-neutrophil cytoplasmic antibody
AUC: Area under the curve
CADI: Chronic allograft damage index
CCR2: MCP-1 receptor
CKD: Chronic kidney disease
eGFR: Estimated glomerular filtration rate
MCP-1: Monocyte chemoattractant protein-1
MMP-2: Matrix metalloproteinase-2
PAI-1: Plasminogen activator inhibitor-1
PIIINP: Amino-terminal propeptide of type III procollagen
STARD: Standards for reporting diagnostic accuracy studies
TG F-β: Transforming growth factor beta
