# Supplementary material for: Biomarkers for the detection of renal fibrosis and prediction of renal outcomes: a systematic review
Source: BMC Nephrol. 2017 Feb 20;18:72. doi: 10.1186/s12882-017-0490-0 (PMC5319065; doi:10.1186/s12882-017-0490-0)
Supplement: Additional file 1: — Study quality scoring system for stage II. Out of the 25 standards for reporting diagnostic accuracy studies (STARD) criteria, we used the ten most relevant parameters to assess quality of studies listed in this review. Studies meeting each criterion are listed under comments on the far right of the table. (DOCX 14 kb) [file 12882_2017_490_MOESM1_ESM.docx]

**Additional file 1: Study quality scoring system for stage II.**

| **Validity criterion** | **Explanation** | **Scoring** | **Comments** |
| --- | --- | --- | --- |
| Participant recruitment | Was recruitment based on presenting symptoms, or results from previous tests? | Presenting symptoms=1  Previous tests= 0 | Based on presenting symptoms in 7 studies (27,28,29,30,32,33,34) |
| Participant sampling | Was its study population a convenience sample or a consecutive series? | Consecutive series=1  Convenience sample or not stated= 0 | Based on convenience sampling in 7 studies (28,29,31,32,33,34,35) |
| Data Collection | Was data collection planned before the index test and reference standard were performed prospectively or retrospectively? | Prospective=1  Retrospective or not stated=0 | Planned and performed prospectively in 8 studies (27,28,29,30,31,32,33,35) |
| Reference standard | Was the definition for the reference standard stated? | Stated=1  Not stated= 0 | Stated in all 9 studies (27,28,29,30,31,32,33,34,35) |
| Materials and methods | Were technical specifications of material and methods stated including how and when measurements were taken? | Stated=1  Not stated=0 | Stated in all 9 studies (27,28,29,30,31,32,33,34,35) |
| Participant characteristics | Were the clinical and demographic characteristics of the study population stated? | Stated=1  Not stated=0 | Stated in all 9 studies  (27,28,29,30,31,32,33,34,35) |
| Blinding | Were readers of the index test and reference standard blinded? | Blinded=1  Not blinded or not stated=0 | Blinding stated in 1 study (30) |
| Completion | Was the number of participants that did not undergo index tests stated (numbers of tests versus sample size stated)? | Stated=1  Not stated=0 | Stated in 6 studies (27,29,30,31,32,35), |
| Diagnostic accuracy and statistical uncertainty | Were estimated of diagnostic accuracy and measures of statistical uncertainty for biomarker results (e.g. 95% confidence intervals) stated? | Stated=1  Not stated=0 | Stated in 8 studies (27,28,30,31,32,33,34,35) |
| Clinical applicability | Was the clinical applicability of biomarker finding stated? | Stated=1  Not stated=0 | Stated in all 9 studies (27,28,29,30,31,32,33,34,35) |
