# Supplementary material for: Biomarkers for the detection of renal fibrosis and prediction of renal outcomes: a systematic review
Source: BMC Nephrol. 2017 Feb 20;18:72. doi: 10.1186/s12882-017-0490-0 (PMC5319065; doi:10.1186/s12882-017-0490-0)
Supplement: Additional file 2: — Methods of fibrosis assessment on biopsy in stage I studies. The table above shows the different methods used to assess fibrosis across different studies in stage I. Fibrosis was evaluated using Banff criteria, image digitalization, numerical quantification score, Oxford classification, morphometric analysis, Lee’s classification, chronic allograft damage index (CADI) score, and semi-quantitatively. (DOC 31 kb) [file 12882_2017_490_MOESM2_ESM.doc]

**Additional file 2: Methods of fibrosis assessment on biopsy in stage I studies**

| **Method of assessing fibrosis** | **Comments** |
| --- | --- |
| Banff | Grade I (<25%), II (26-50%), III (>50%) |
| Image digitalization | Image Pro Plus programs create digitalized images out of which five randomly selected fields are evaluated for fibrosis and expressed in micrometers. |
| Numerical quantification software | Trichome stained sections are analyzed using color segmentation image analysis software for quantification of interstitial fibrosis. |
| Oxford Classification | T0 (<25%), T1 (26-50%), T2 (>50%) |
| Morphometric analysis | Point counting analysis |
| Lee’s Classification | Grade I (none), II (<25%), III (25-49%), IV (50-75%), V (>75%) |
| CADI score | Score 1 (<25%), 2 (26-50%), 3(>50%) |
| Semi-quantitatively | Visual assessment using trichome stained slides (standard assessment) |

CADI: chronic allograft damage index.
