# Supplementary material for: Biomarkers for the detection of renal fibrosis and prediction of renal outcomes: a systematic review
Source: BMC Nephrol. 2017 Feb 20;18:72. doi: 10.1186/s12882-017-0490-0 (PMC5319065; doi:10.1186/s12882-017-0490-0)
Supplement: Additional file 3: — Patient populations and renal outcomes assessed in stage I and stage II of the systematic review. The above table shows the heterogeneity in the data with varying patient populations and different operational definitions of worsening renal function. (DOC 43 kb) [file 12882_2017_490_MOESM3_ESM.doc]

Additional file 3: Patient populations and renal outcomes assessed in stage I and stage II of the systematic review

| **Stage I references** | **Patient population** |  | **Stage II references** | **Patient population** | **Renal Outcome** |
| --- | --- | --- | --- | --- | --- |
| El Ghoul *et al* [14] | Biopsies for clinical reasons |  | Chen *et al* [29] | Unilateral ureteral obstruction requiring percutaneous nephrostomy | Non-functioning kidney group defined by no improvement in eGFR. |
| Teppo *et al* [15] | Transplant recipients |  | Harris *et al* [30] | Renal transplant recipients | Biopsy proven chronic allograft nephropathy using Banff 97 |
| Soylemezoglu *et al* [16] | Biopsies for clinical reasons |  | Wong *et al* [31] | Type II diabetes | Doubling of serum creatinine |
| Honkanen *et al* [20] | Idiopathic membranous glomerulonephritis |  | Hsu *et al* [27] | Non diabetic patients referred for coronary angiography | eGFR decline >25% from baseline. |
| Susianti *et al* [21] | Lupus nephritis |  | Shi *et al* [28] | Chronic tubulointerstitial nephropathy | Continuous outcome of eGFR decline |
| Murakami *et al* [22] | Glomerulopathy |  | Titan *et al* [32] | Macroalbuminuric type II diabetes | Composite outcome of risk of dialysis, or doubling of serum creatinine or death. |
| Zhang *et al* [24] | Lupus nephritis |  | Verhave *et al* [33] | Diabetic nephropathy | The rate of eGFR decline as a continuous outcome |
| Chang *et al* [25] | Transplant recipients |  | Ogliari *et al* [34] | Simultaneous Pancreas Kidney recipients | Graft Loss |
| Sanders *et al* [26] | Anti-neutrophil cytoplasmic antibodies vasculitis |  | Nadkarni *et al* [35] | Type II diabetes | eGFR decline >40% from baseline |
| Grenzi *et al* [18] | Transplant  Recipients |  |  |  |  |
| Amer *et al* [10] | Transplant recipients |  |  |  |  |
| Barbosa de Deus  *et al* [11] | Glomerulopathy |  |  |  |  |
| Pallet *et al* [12] | Biopsies for clinical reasons |  |  |  |  |
| Zhu *et al* [13] | IgA nephropathy |  |  |  |  |
| Metalidis *et al* [17] | Transplant recipients |  |  |  |  |
| Liu *et al* [19] | IgA nephropathy |  |  |  |  |
| Lu *et al* [23] | IgA nephropathy |  |  |  |  |
